# Supplementary material for: The interactome of intact mitochondria by cross-linking mass spectrometry provides evidence for coexisting respiratory supercomplexes
Source: Mol Cell Proteomics. 2017 Dec 8;17(2):216–32. doi: 10.1074/mcp.RA117.000470 (PMC5795388; doi:10.1074/mcp.RA117.000470)
Supplement: Supplemental Data [file supp_RA117.000470_133922_0_supp_23978_fzffwf.zip › spectra_annotation/mito_DR_spectra_annotation/149-1-10-1-10-1.pdf]

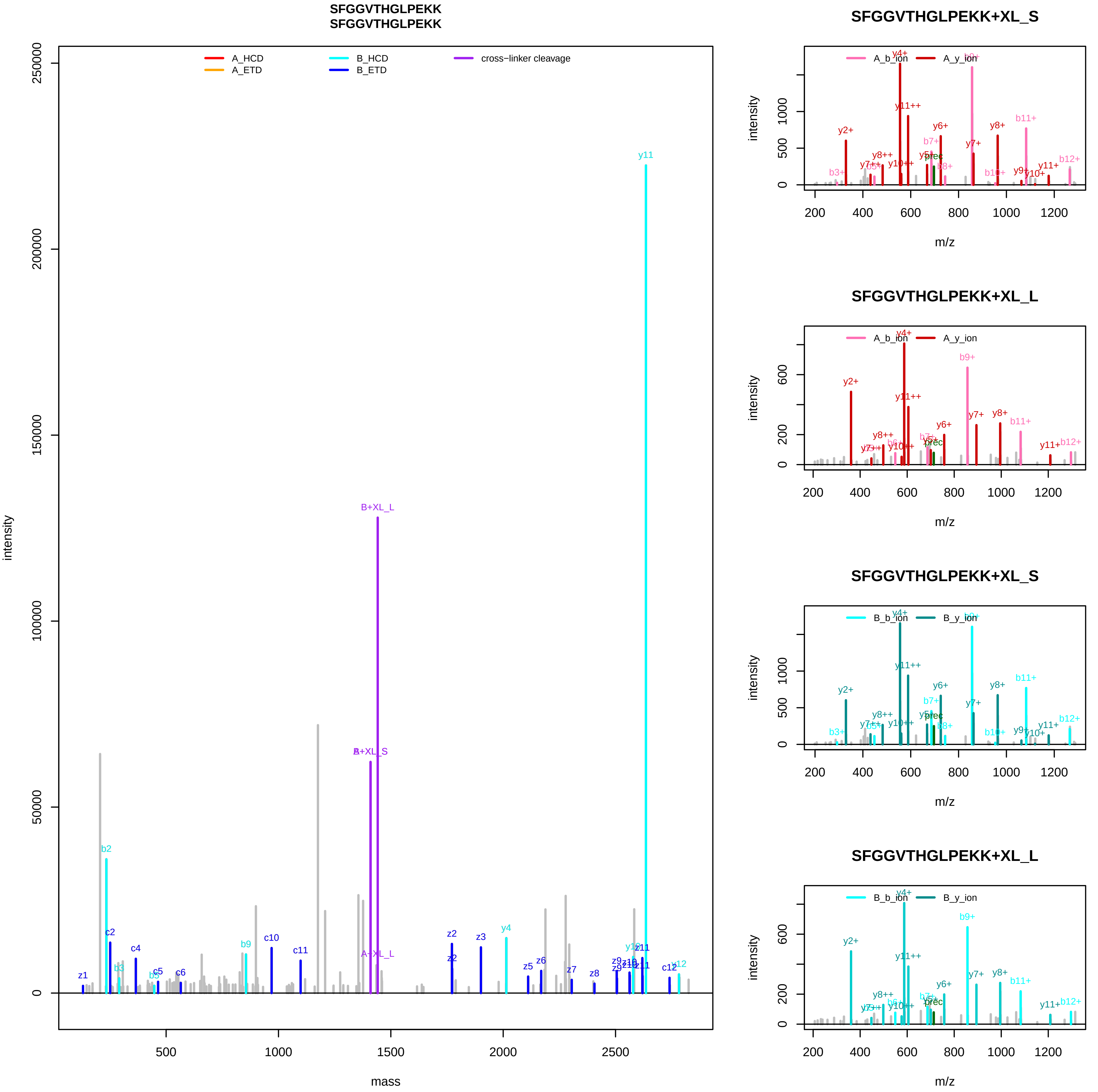
SFGGVTHGLPEKK
SFGGVTHGLPEKK
A_HCD
A_ETD
B_HCD
B_ETD
cross−linker cleavage
intensity
mass
y11
B+XL_L
A+XL_S
A+XL_L
b2
c2
b3
c4
b5
c5
c6
b9
c10
c11
z1
z2
z3
y4
z5
z6
z7
z8
z9
z10
z11
y12
c12
SFGGVTHGLPEKK+XL_S
SFGGVTHGLPEKK+XL_L
A_b_ion
A_y_ion
B_b_ion
B_y_ion
intensity
m/z
y2+
y4+
y6+
y7+
y8+
y8++
y9+
y10+
y10++
y11+
y11++
b3+
b5+
b6+
b7+
b8+
b9+
b10+
b11+
b12+
prec
